# Combined systemic inflammatory immunity index and prognostic nutritional index scores as a screening marker for sarcopenia in patients with locally advanced gastric cancer

**DOI:** 10.3389/fnut.2022.981533

**Published:** 2022-08-15

**Authors:** Ping'an Ding, Jingxia Lv, Chenyu Sun, Shuya Chen, Peigang Yang, Yuan Tian, Qin Zhou, Honghai Guo, Yang Liu, Qun Zhao

**Affiliations:** ^1^The Third Department of Surgery, The Fourth Hospital of Hebei Medical University, Shijiazhuang, China; ^2^Hebei Key Laboratory of Precision Diagnosis and Comprehensive Treatment of Gastric Cancer, Shijiazhuang, China; ^3^AMITA Health Saint Joseph Hospital Chicago, Chicago, IL, United States; ^4^Newham University Hospital, London, United Kingdom; ^5^Radiation Oncology, Mayo Clinic, Rochester, MN, United States

**Keywords:** systemic immune-inflammatory index, prognostic nutritional index, locally advanced gastric cancer, sarcopenia, marker

## Abstract

**Background:**

Sarcopenia is associated with poor clinical outcomes in patients with locally advanced gastric cancer (LAGC). Currently, the diagnostic criteria for sarcopenia are complex and laborious. Increased evidence suggests the inflammatory state of the body is closely associated with the development of sarcopenia. The systemic immune-inflammatory index (SII) and the prognostic nutritional index (PNI) are representative blood indicators of the status of the systemic inflammatory response, but the clinical significance of the combined testing of these two indicators remains unclear. We aimed to develop a simple and practical risk score (SII-PNI score) to screen patients with LAGC for sarcopenia on admission for early diagnosis.

**Methods:**

We registered a prospective clinical study from January 2011 to May 2016 involving 134 patients with LAGC undergoing radical surgical resection. All patients followed the definition of sarcopenia in the Asian Working Group on Sarcopenia (AWGS) guidelines and were divided into sarcopenia and non-sarcopenia groups. SII-PNI score 0–2 was scored as 2 for high SII (≥432.9) and low PNI ( ≤ 49.5); score 1, either high SII or low PNI; score 0, no high SII or low PNI.

**Results:**

All patients underwent radical surgery, including 31 patients (23.13%) with sarcopenia according to AWGS criteria. The SII-PNI score was significantly lower in the non-sarcopenic patients than in the sarcopenic patients (*p* < 0.001). Logistic multivariate analysis showed that the SII-PNI score predicted an independent prognostic factor for sarcopenia (*p* < 0.001). Patients with high SII-PNI scores had significantly worse prognosis than those with low SII-PNI scores (*p* < 0.001). The SII-PNI score was an independent prognostic factor for predicting overall survival and disease-free survival (*p* = 0.016, 0.023).

**Conclusion:**

Peripheral blood parameters SII-PNI scores accurately identify sarcopenia in patients with LAGC and could be used as potential systemic markers.

12pt

## Introduction

Gastric cancer is a common malignant tumor of the digestive tract, ranking fifth in incidence and fourth in mortality among malignant tumors (1). Most patients with gastric cancer are at an advanced stage at initial diagnosis and despite advances in current anti-tumor treatments including surgery, chemotherapy, targeted and immunotherapy, the overall prognosis remains poor with only slight improvement (2–4). Apart from the prognosis of patients with gastric cancer, which is closely related to tumor-specific factors (pathological type, tumor stage, etc.), the nutritional status and skeletal muscle mass (SMM) of patients are also important factors (5, 6).

Cancer cachexia is a syndrome of progressive loss of skeletal muscle mass, anorexia, systemic inflammation, and other metabolic abnormalities that lead to decreased bodily function (7, 8). Sarcopenia is an age-related syndrome characterized by progressive loss of skeletal muscle mass and function (8, 9). Numerous studies have shown that sarcopenia is not only high in the elderly population, but also has a high incidence in cancer patients (10, 11). The incidence of sarcopenia in patients with gastric cancer is as high as 28.8%, and especially in patients with locally advanced gastric cancer (LAGC) may be higher (12, 13). Concomitantly, sarcopenia in patients with LAGC has received widespread attention in recent years, as it is not only associated with increased adverse outcomes such as post-operative complications, but also has a detrimental effect on long-term survival (14–16). Thus, early diagnosis of sarcopenia in clinical practice is an important part of the management of LAGC patients. Currently, based on international consensus (17–19), the diagnosis of sarcopenia requires muscle mass [e.g., computed tomography (CT), dual-energy x-ray absorptiometry (DXA)], muscle strength [e.g., handgrip strength (HGS) and walking speed]. However, these complex procedures in a busy clinical setting limit sarcopenia screening and are not readily available. Therefore, there is an urgent need for a simple, reproducible, accurate and cost-effective biomarker to screen for and predict sarcopenia.

In recent years, an increasing number of studies have found that inflammatory factors in the body promote muscle atrophy, stimulate protein catabolism and inhibit muscle synthesis (20, 21). A meta-analysis by Bano et al. (22) of 17 studies included on the relationship between sarcopenia and the inflammatory response showed that C-reactive protein (CRP) levels were significantly higher in those with sarcopenia than in those in the non-sarcopenic group. The same results were obtained in two other studies, which confirmed that high levels of inflammatory factors were negatively associated with muscle strength and mass (23, 24). Growing evidence indicated that the inflammatory response is closely related to the occurrence and development of sarcopenia (25, 26). The increase of inflammatory cytokines in the body promotes the increase of muscle metabolism, which will further induce the imbalance of muscle protein synthesis and catabolism (27, 28). The systemic inflammatory immunity index (SII) is a new inflammatory index based on peripheral blood neutrophil, lymphocyte and platelet counts that adequately reflects the balanced relationship between immunity and inflammation (29, 30). A recent cross-sectional study that included 4,224 elderly patients found that higher SII levels were associated with an increased prevalence of sarcopenia (31). Meanwhile, Bullock et al. found that the prognostic nutritional index (PNI) calculated by peripheral blood albumin and lymphocyte count can be seen as a marker of inflammation rather than nutrition, which can be used as a tool for screening malnutrition and sarcopenia (32). Nonetheless, the value of SII combined with PNI in screening for sarcopenia in patients with LAGC has not been reported.

Based on the above reasons, we are wondering whether we can explore the prediction of muscle mass in LAGC patients by SII combined with PNI and determine the optimal cut-off value for SII combined with PNI to predict sarcopenia and its impact on the prognosis of LAGC patients.

## Materials and methods

### Patients and study design

This study is a prospective observational study that included 290 LAGC patients receiving surgery in the Fourth Hospital of Hebei Medical University from January 2011 and May 2016. This study is approved by the Ethics Committee of the Fourth Hospital of Hebei Medical University (Approval Number: 20111214029). Inclusion criteria were as follows: (I) pathology conformed the gastric cancer diagnosis; (II) age ≥ 18 years; (III) no other anti-tumor therapy before surgery; (IV) the Eastern Cooperative Oncology Group (ECOG) activity status score was ≤ 2 points. The exclusion criteria were as follows: (I) residue of cancer cells in surgical margin (R1/R2 resection); (II) incomplete clinical data; (III) combined with other tumor history or hematological diseases; (IV) preoperative combined infection leads to abnormal blood routine results; (V) the presence of metal implants in the lumbar spine prevents measurement of skeletal muscle at L3 level.

### Laboratory measurements

Peripheral venous blood was collected from all patients on an empty stomach within 1 week before surgery. Peripheral blood neutrophil, lymphocyte, and platelet counts in LAGC patients were analyzed by the method in our previous study (29, 30). The specific operation process is as follows: using an automatic hematology analyzer (Beckman Coulter LH750) to measure and analyze the counts of neutrophils, platelets and lymphocytes, and the albumin level using an automatic hematology analyzer (Beckman Coulter AU5800) assay analysis. The SII was defined as follows: SII = P × N/L, where P, N, and L were the platelet, neutrophil, and lymphocyte counts, respectively (29, 30, 33). Similarly, PNI is calculated by albumin plus 5 times lymphocyte count (34).

### Measurement of muscle mass and strength

Preoperative abdominal and pelvic CT images of all enrolled LAGC patients were analyzed, and skeletal muscle area at the L3 level of the lumbar spine was measured. The images of each patient were uploaded to the picture archiving and communication system (PACS, SIEMENS SOMATOM) for processing. The following tissue Hounsfield Units (HU) thresholds were used: skeletal muscle attenuation ranged from −29 to 150 HU (35). The software evaluates and measures the pixel area of the corresponding area of skeletal muscle attenuation to obtain the skeletal muscle area (SMA). The skeletal muscle index (SMI) was then calculated by dividing the SMA by the square of the patient's height. Meanwhile, all patients were assessed for handgrip strength (HGS) using a Camry dynamometer (EH101; Xiangshan Company, Guangdong, China) before surgery. Two measurements were made alternately, and the maximum value was used for further analysis.

### Diagnostic criteria for sarcopenia

The definition of sarcopenia in this study was assessed using the Asian Working Group on Sarcopenia (AWGS) (18) consensus. We used the following cut-off values to define sarcopenia: SMI <40.8 cm^2^/m^2^ in men and SMI <34.9 cm^2^/m^2^ in women as muscle loss, and HGS <26 kg in men and HGS <18 kg in women as sarcopenia.

### Follow-up of participants

Overall survival (OS) was defined as the time interval from the start of initial recruitment to cancer-related death or final contact. Disease-free survival (DFS) is defined as the time from the start of randomization until disease recurrence or death of the patient due to disease progression. All patients were recommended to have an enhanced CT scan of the abdomen and pelvis every 3 months for the first 3 years post-operatively and every 6 months for the 4th to 5th post-operative years. Follow-up methods mainly included telephone encounters, outpatient visits, and hospitalization.

### Statistical analysis

All statistical analyses were performed using IBM SPSS Statistics for Windows, version 26.0 (IBM Corp, Armonk, NY, USA) and MedCalc Statistical Software v15.2 (MedCalc Software bvba, Ostend, Belgium). Categorical variables were expressed as numbers and proportions, using the *X*^2^-test or Fisher's exact test. Continuous variables were expressed as mean with standard deviation (SD) or median with interquartile range (IQR). All continuous data in this study were non-normally distributed according to the Kolmogorov-Smirnov test, so the Wilcoxon rank-sum test was used to compare differences between groups. Scatter plots and Pearson's correlation coefficient were used to assess linear correlations. Multivariate logistic regression was used to analyze the effect of various factors on the prevalence of sarcopenia. The subject receiver operating characteristic (ROC) curve and area under the curve (AUC) were used to assess the ability of the SII and PNI to screen for sarcopenia. Kaplan-Meier curves were performed to estimate OS and DFS, and a log-rank test was used to compare the difference between groups. Cox regression models were used to identify independent prognostic factors. A *p*-value <0.05 was considered to show statistical significance.

## Results

### Patients characteristics

**Figure 1:**
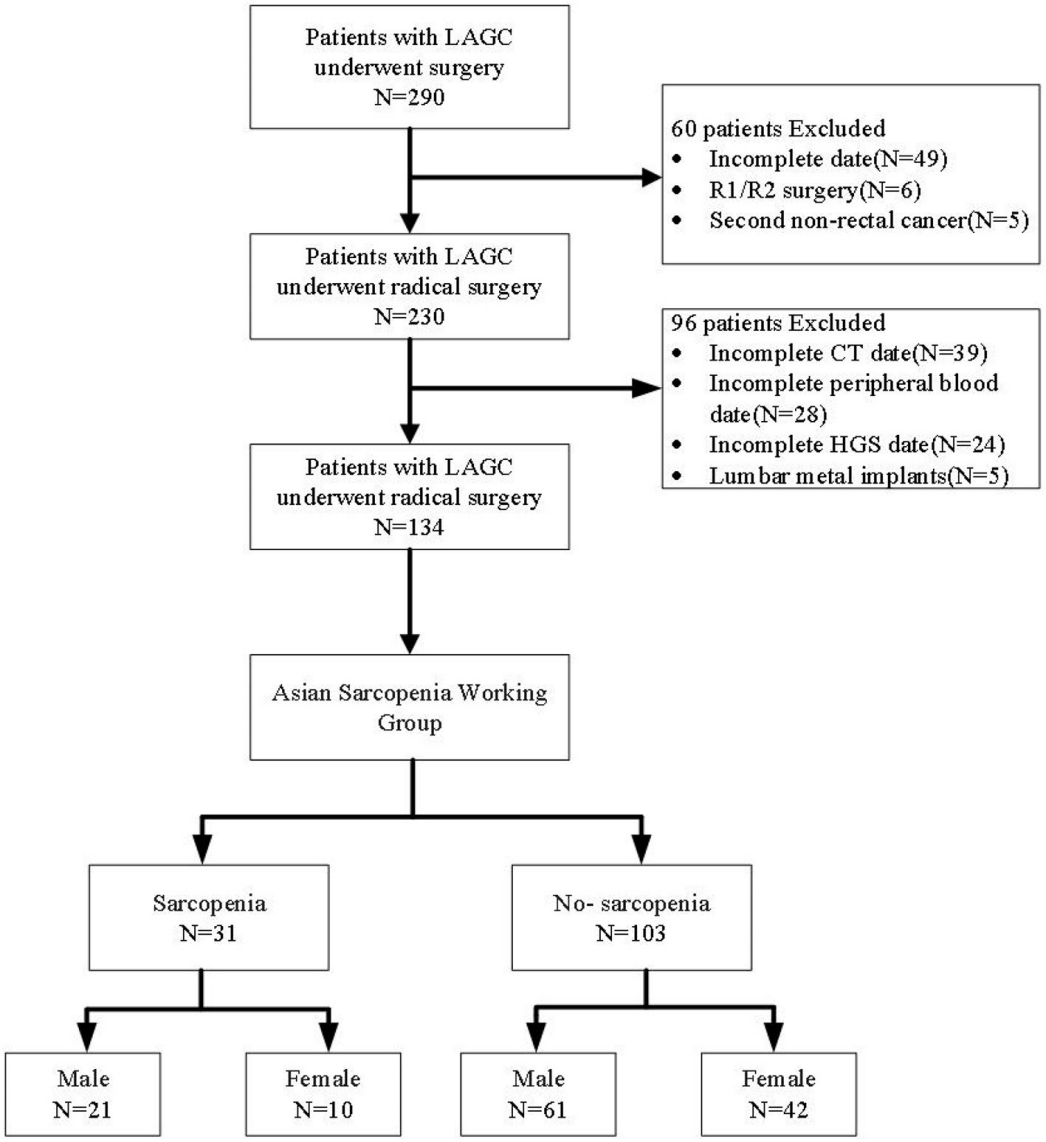
Flow diagram participants included in the study. LAGC, locally advanced gastric cancer; HGS, handgrip strength.

**Figure 2:**
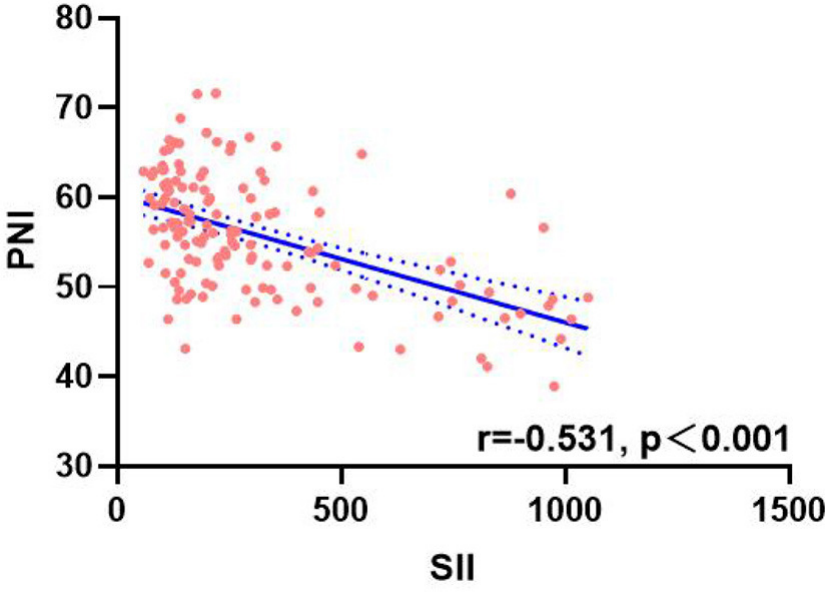
Correlation analysis between SII and PNI. SII, systemic inflammatory immunity index; PNI, prognostic nutritional index.

**Figure 3:**
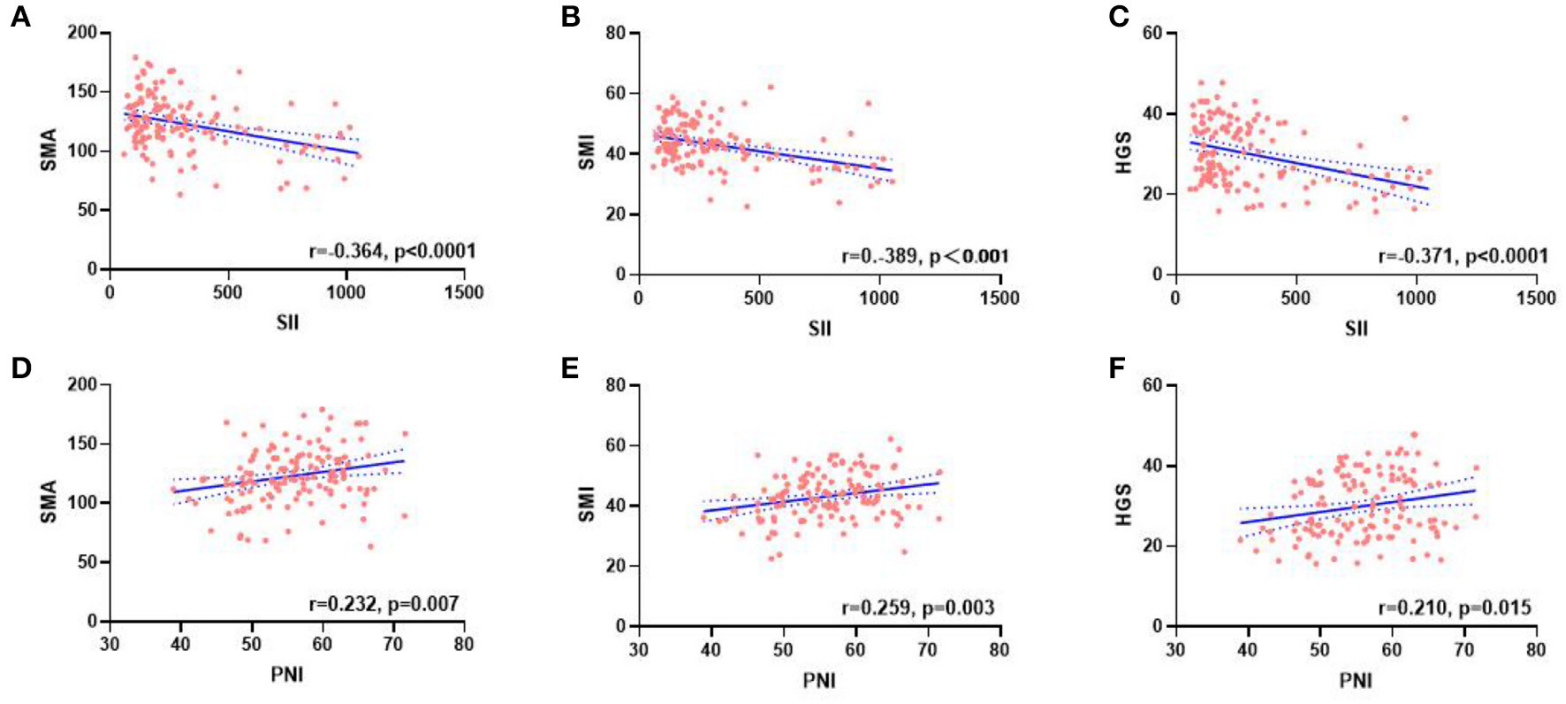
Correlation between the SII **(A–C)**, PNI **(D–F)**, and SMA, SMI, and HGS. SII, systemic inflammatory immunity index; PNI, prognostic nutritional index; SMA, skeletal muscle area; SMI, skeletal muscle mass index; HGS, handgrip strength.

A total of 134 patients with LAGC were included according to the inclusion and exclusion criteria of this study (Figure 1). The patients' characteristics are presented in Table 1. The mean age was 58.4 years were enrolled in this study and a total of 82 male (61.19%) patients with LAGC. According to the AWGS consensus definition of sarcopenia, there were 21 sarcopenia patients (25.61%) out of 82 men, and 10 sarcopenia patients (19.23%) out of 52 women among the patients. The median SII before radical surgery for all patients was 217.3, ranged from 59.1 to 1,051.2 and in addition for PNI the median value was 55.9, ranged from 38.9 to 71.6. Meanwhile, the two systemic indices SII and PNI had close negative correlation (*r* = −0.531, *p* < 0.001; Figure 2). As shown in Figure 3, SII had a negative correlation with SMA (*r* = −0.364; *p* < 0.001), SMI (*r* = −0.389; *p* < 0.001), and HGS (*r* = −0.371; *p* < 0.001), while PNI had a positive correlation with SMA (*r* = 0.232; *p* = 0.007), SMI (*r* = 0.259; *p* = 0.003), and HGS (*r* = 0.210; *p* = 0.015).

**Table 1:** Demographic and clinical characteristics of the patients with cancer.

**Variables**	**All patients (*N* = 134)**	**Male (*N* = 82)**	**Female (*N* = 52)**
**Age, years**	58.4 ± 8.7	58.3 ± 8.3	58.6 ± 9.3
**BMI, kg/m** ^**2**^	23.2 ± 3.0	22.9 ± 2.9	23.7 ± 3.1
**Tumor location**, ***n*** **(%)**			
Up 1/3	49 (36.57)	32 (39.02)	17 (32.69)
Middle 1/3	24 (17.91)	11 (13.41)	13 (25.00)
Low 1/3	61 (45.52)	39 (47.56)	22 (42.31)
**Tumor size (cm)**	5.3 ± 3.1	5.2 ± 2.9	5.3 ± 2.8
**pTNM stage**, ***n*** **(%)**			
II	36 (26.87)	26 (31.71)	10 (19.23)
III	98 (73.13)	56 (68.29)	42 (80.77)
**Handgrip strength (Kg)**	29.9 ± 8.0	33.9 ± 7.1	23.7 ± 4.7
**Albumin (g/L)**	42.4 ± 3.8	41.9 ± 3.8	43.5 ± 3.5
**Prealbumin (mg/L)**	213.8 ± 23.4	210.8 ± 26.3	214.1 ± 29.6
**Total protein (g/L)**	69.7 ± 17.1	68.9 ± 18.0	70.2 ± 19.4
**SMA (cm** ^**2**^ **)**	123.2 ± 23.5	129.5 ± 20.7	113.2 ± 24.2
**SMI (cm** ^**2**^ **/m** ^**2**^ **)**	43.1 ± 7.5	44.2 ± 6.5	41.4 ± 8.7
**Neutrophil counts, 10** ^**9**^ **/L**	3.5 ± 1.5	3.6 ± 1.5	3.2 ± 1.4
**Platelet counts, 10** ^**9**^ **/L**	196.9 ± 68.7	197.5 ± 66.9	195.9 ± 72.0
**Lymphocyte counts, 10** ^**9**^ **/L**	2.9 ± 1.1	2.6 ± 1.0	2.8 ± 1.3
**SII**	312.6 ± 253.4	330.6 ± 258.5	284.3 ± 244.9
**PNI**	55.8 ± 6.8	55.0 ± 6.7	57.0 ± 6.8
**Sarcopenia**, ***n*** **(%)**			
Yes	31 (23.13)	21 (25.61)	10 (19.23)
No	103 (76.87)	61 (74.39)	42 (80.77)

*BMI, body mass index; HGS, handgrip strength; SMA, skeletal muscle mass; SMI, skeletal muscle index; SII, systemic inflammatory immunity index; PNI, prognostic nutritional index. Values are presented as mean (SD) unless otherwise noted*.

### Optimal cut-off values for SII and PNI values for pre-surgical diagnosis of sarcopenia

**Figure 4:**
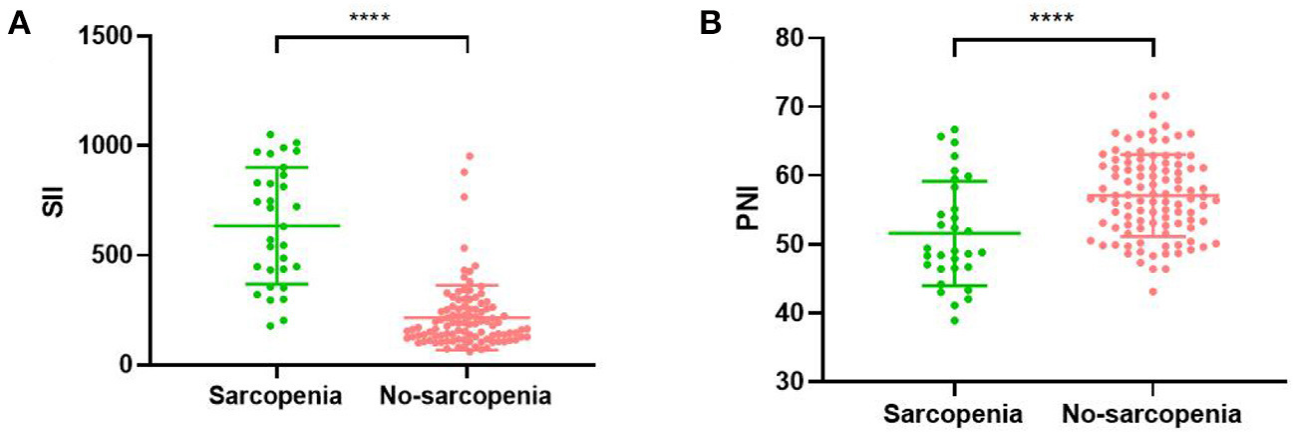
Relationship between sarcopenia and the SII **(A)**/PNI **(B)**. SII, systemic inflammatory immunity index; PNI, prognostic nutritional index; **** <0.001.

**Figure 5:**
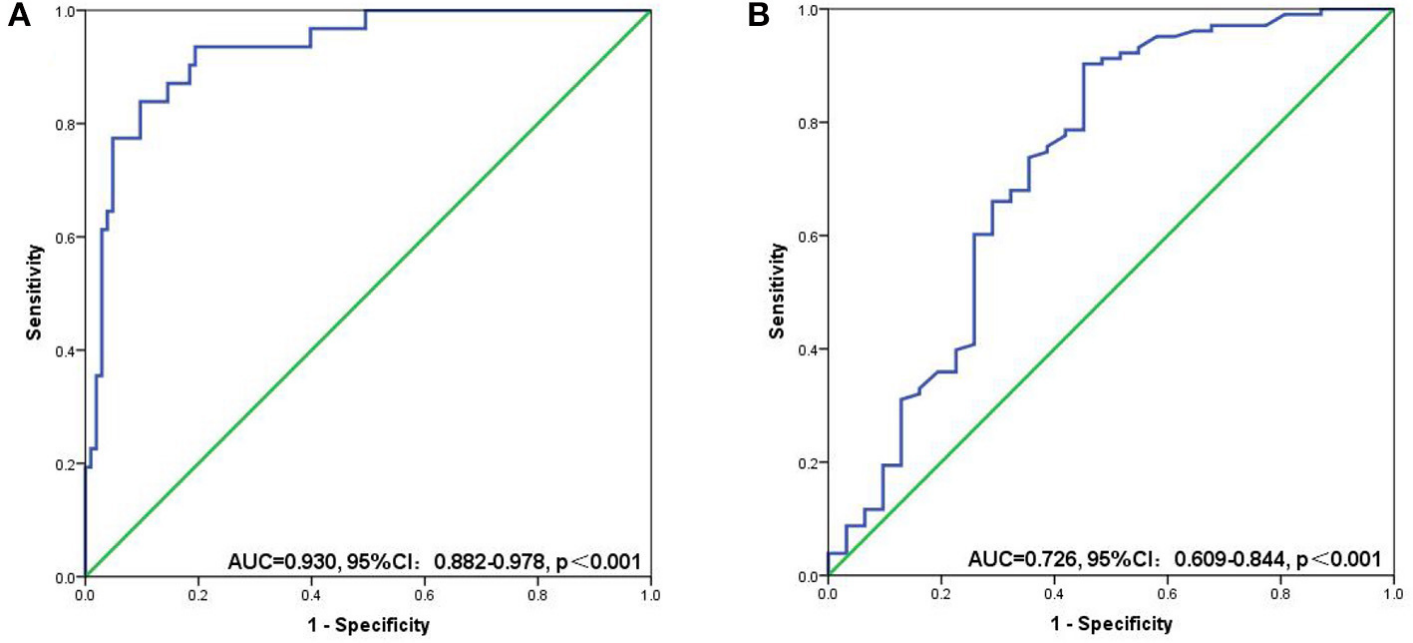
ROC curves for discriminating patients with sarcopenia and those with non-sarcopenia according to values of the SII **(A)** and PNI **(B)**. SII, systemic inflammatory immunity index; PNI, prognostic nutritional index.

The mean SII values were significantly lower in the non-sarcopenic patients than in the sarcopenic patients (215.7 ± 148.0 vs. 634.7 ± 266.0, *p* < 0.001) (Figure 4A). However, patients in the non-sarcopenic group had higher mean PNI values than those in the sarcopenic group (57.1 ± 6.0 vs. 51.6 ± 7.6, *p* < 0.001) (Figure 4B). We used the Youden-Index to maximize the “sensitivity + specificity-1” value to calculate serum SII and PNI cut-off values of 432.9 (AUC = 0.930, 95%CI: 0.882–0.978, *p* < 0.001, sensitivity: 0.774, specificity: 0.951) and 49.5 (AUC = 0.726, 95%CI: 0.609–0.844, *p* < 0.001, sensitivity: 0.903, specificity: 0.548), respectively (Figure 5). Patients were divided into three groups according to the optimal cut-off values for SII and PNI: high SII (≥432.9) and low PNI ( ≤ 49.5) were defined as a score of 2 (*n* = 17, 12.69%); either high SII or low PNI was defined as a score of 1 (*n* = 21, 15.67%); and a score of 0 (*n* = 96, 71.64%) with no high SII or low PNI.

### Associations between SII-PNI score and sarcopenia

Seven patients (7.29%) with SII-PNI score of 0 were diagnosed as sarcopenia, while 7 patients (33.33%) with SII-PNI score of 1 and 17 patients (100.00%) with SII-PNI score of 2 were diagnosed as sarcopenia, and the differences among the three groups were significant (*p* < 0.001). To further analyze the association between the SII-PNI score and the presence of sarcopenia, we used logistic regression analysis to identify predictors of sarcopenia. Univariate analysis showed that SII-PNI score, age, BMI, preoperative hemoglobin, tumor size, and pTNM stage were risk factors for sarcopenia. After adjusting for age, anemia, and tumor size, multivariate analysis showed that SII-PNI score (OR = 26.214, 95%CI: 11.923–87.212, *p* < 0.001) was still an independent predictor of increased risk of sarcopenia (Table 2).

**Table 2:** Univariate and multivariate analyses the risk of sarcopenia.

**Factors**	**Univariable analysis**		**Multivariate analysis**	
	**OR (95% CI)**	***p-*value**	**OR (95% CI)**	***p-*value**
**Age, years**		0.009		0.321
≤ 58	Reference		Reference	
>58	3.636 (1.376–9.607)		1.561 (0.140–6.827)	
**Sex**		0.395		
Male	Reference			
Female	1.446 (0.618–3.381)			
**BMI, kg/m** ^**2**^		0.027		0.001
≥18.5	Reference		Reference	
<18.5	4.760 (1.193–18.994)		5.121 (2.223–10.537)	
**Anemia**		0.043		0.332
No	Reference		Reference	
Yes	2.589 (1.029–6.515)		1.621 (0.621–4.612)	
**Tumor location**		0.963		
Up 1/3	Reference			
Middle 1/3	1.023 (0.341–2.622)			
Low 1/3	0.794 (0.081–4.423)			
**Tumor size, cm**		0.024		0.118
<5.0	Reference		Reference	
≥5.0	2.642 (1.137-6.137)		1.435 (0.652-3.232)	
**pTNM stage**		0.021		0.012
II	Reference		Reference	
III	4.400 (1.247–15.521)		2.432 (1.716–8.731)	
**SII-PNI score**		<0.001		<0.001
0	Reference		Reference	
1	6.357 (1.935–20.888)		7.237 (3.245–33.341)	
2	41.667 (22.793–132.530)		26.214 (11.923–87.212)	

*BMI, body mass index; SII, systemic inflammatory immunity index; PNI, prognostic nutritional index*.

### The relationship between SII-PNI score and prognosis

**Figure 6:**
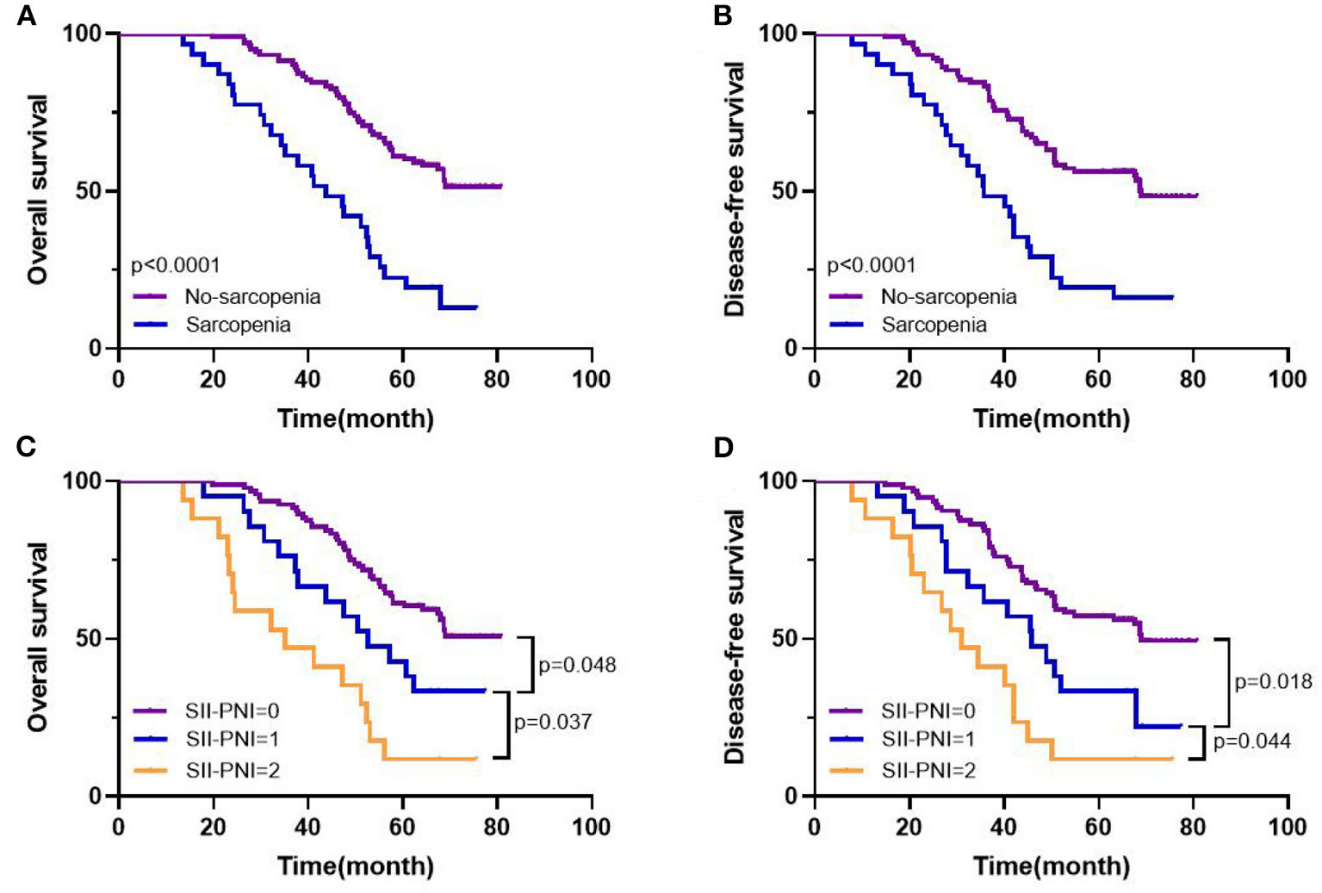
Kaplan-Meier survival curves in patients with LAGC. 5-year overall survival **(A)**, disease-free survival **(B)** based on sarcopenia status; 5-year overall survival **(C)**, disease-free survival **(D)** based on SII-PNI score.

All patients were followed up, and the median follow-up time was 55.7 months (range: 13.6–80.8 months). The 5-year OS of the whole group of patients was 52.99%, and the 5-year DFS was 47.76%. Further subgroup analysis found that the 5-year OS (61.17% vs. 25.81%, *p* < 0.001) and DFS (56.31% vs. 19.35%, *p* < 0.001) of non-sarcopenic patients were better than those of sarcopenic patients, and the difference was statistically significant (Figures 6A,B). Furthermore, based on the different SII-PNI scores, we found that the 5-year OS and DFS of patients with 0 score were 61.46% and 57.29%, respectively, while the 5-year OS of patients with 1 score and 2 score were 42.86% and 17.65%, respectively, and the 5-year DFS were 33.33% and 11.76%, respectively, and there were significant differences in 5-year OS and DFS among the three groups (all *p* < 0.05) (Figures 6C,D). The result of Cox regression model revealed that SII-PNI scores was an independent prognostic factor of OS (adjusted HR = 2.422, 95%CI: 1.022–6.727, *p* = 0.016) and DFS (adjusted HR = 2.62, 95%CI: 1.341–5.247, *p* = 0.023) in patients with LAGC.

## Discussion

Our study showed that SII was negatively correlated with muscle mass and hand grip strength in LAGC patients, while PNI was positively correlated. Furthermore, both indicators performed satisfactorily and comparably in predicting sarcopenia. Moreover, we calculated separate cut-off values for the SII and PNI diagnoses of sarcopenia, and based on these cut-off values the two indicators were combined to form a new SII-PNI score. As the SII-PNI score increased, the possibility of sarcopenia is also greater, indicating that SII-PNI score has high predictive value in predicting sarcopenia. These results suggest that the SII-PNI score can be used as a surrogate biomarker to assess sarcopenia in patients with LAGC, with a score of 2 essentially determining that sarcopenia will develop in patients. Alternatively, based on the results of the survival curves, we found that the SII-PNI score can also predict 5-year OS and DFS in LAGC patients and can also be used as a predictive marker for survival prognosis.

Numerous studies have found that the essence of sarcopenia is the decline of muscle fibers and the degradation of proteins, which is closely associated with increased inflammation, oxidative stress damage, mitochondrial dysfunction, abnormal cellular autophagy and dysregulation of muscle mass regulatory factors (36, 37). Previous studies on markers of sarcopenia have mostly focused on serum creatinine and cystatin C, but also on related inflammatory mediators such as C-reactive protein (CRP) (23), tumor necrosis factor-alpha (TNF-α) (38), and interleukins (IL) (22). Currently, screening for these markers requires additional cost to the patient and redundant targeted testing. As a highly accessible and reproducible biomarker, the association of PNI nutritional status indicators and SII inflammatory indicators with prognosis in patients with various tumors, including gastric cancer, has received increasing attention (39–41). However, few studies have focused on the relationship between SII and PNI on sarcopenia.

To the best of our knowledge, this is the first analysis of the association of the SII-PNI score with sarcopenia. A previous retrospective study found that sarcopenia was significantly associated with neutrophil-to-lymphocyte Ratio (NLR) in elderly patients with esophageal squamous cell carcinoma (ESCC), and both were independent prognostic factors in ESCC patients (21). Another multicentre prospective study found that NLR, PNI, SII and platelet lymphocyte ratio (PLR) were all good predictors of sarcopenia, with ALI [BMI (kg/m^2^) × albumin (g/dl)/NLR] being the best predictor (20, 21). These studies have shown that all of these indicators based on inflammation can be used as markers to predict sarcopenia. The present study combined the strengths and weaknesses of markers from previous studies, combining neutrophil, platelet and lymphocyte counts and albumin levels to form the SII-PNI score, and based on this score found that patients with higher scores were more likely to develop sarcopenia.

Recent studies have found that high levels of inflammatory factors promote muscle atrophy, stimulate increased proteolytic metabolism and inhibit muscle synthesis, so inflammatory factors are negatively associated with muscle strength and mass (20, 21, 42–44). Similarly, other studies have found that increased inflammatory factors can also lead to insulin resistance and muscle wastage through activation of the ubiquitin-proteasome protein hydrolysis pathway, and that muscle wastage itself further exacerbates insulin resistance (45). Meanwhile, a number of studies have found that the inflammatory response is also an independent risk factor for the prognosis of patients with LAGC (46, 47). A high inflammatory response leads to the release of a series of inflammatory mediators from tumor cells, causing oxidative damage and DNA mutation, which in turn alters the tumor microenvironment and promotes the proliferation and migration of tumor cells (47, 48). The systemic inflammatory response will aggravate malnutrition and decline in body function of patients with malignant tumors, which will promote poor prognosis of patients with malignant tumors (29, 30). It has been reported that muscle hormones secreted by myocytes can inhibit the growth of tumor cells, and that muscle hormone expression may be reduced in sarcopenia, leading to tumor proliferation and recurrence (49). In addition, the systemic inflammatory response will release more pro-inflammatory cytokines and growth factors, resulting in profound catabolic effects on the body's metabolism, ultimately leading to increased muscle breakdown (50). Low muscularity could contribute to local inflammation in the muscle, leading to further breakdown and driving systemic inflammation (8). In conclusion, systemic inflammation, tumor invasion and proliferation and sarcopenia are closely related, forming a vicious circle. In our study, there were significant differences in prognosis between patients with different SII-PNI scores, and also between patients with and without sarcopenia. Systemic inflammation and malnutrition are unavoidable problems for cancer patients. Therefore, there is an urgent need to enhance the patient's comprehensive treatment to prevent muscle wastage and improve physical condition, stamina and quality of life.

It is noteworthy that a few limitations of current research also exist. First, this was a prospective with a small sample size and the selection of LAGC patients may have been biased, which would have limited the generalizability of the results. Second, this study did not include patients with LAGC from other centers to validate the diagnostic efficacy of the SII-PNI score for sarcopenia. Given this, there is an urgent need for a larger, multicentre prospective study to explore the predictive power of the SII-PNI score for sarcopenia to consolidate our findings.

## Conclusions

In conclusion, this study suggests that the SII-PNI score may be a simple, cost-effective, and efficient screening tool for sarcopenia in LAGC patients. Furthermore, a higher SII-PNI score is associated with poorer 5 year OS and DFS, indicating its promising prognostic value for long-term survival. However, further studies with larger sample size and different patient groups are required to validate these findings.

## Data availability statement

The raw data supporting the conclusions of this article will be made available by the authors, without undue reservation.

## Ethics statement

This study is registered with ClinicalTrials.gov, Registry Number NCT01516944, and approved by the Ethics Committee of the Fourth Hospital of Hebei Medical University (Approval Number: 20111214029). The patients/participants provided their written informed consent to participate in this study.

## Author contributions

Conception and design and administrative support: QZha. Provision of study materials or patients: PD, PY, YT, HG, and JL. Collection and assembly of data: PD, PY, YT, and HG. Data analysis and interpretation: PD, CS, SC, and QZha. All authors writing and gave final approval of the manuscript.

## Funding

This work was supported by the Cultivating Outstanding Talents Project of Hebei Provincial Government Fund (No. 2019012), the Hebei Public Health Committee County-Level Public Hospitals suitable health technology promotion and storage project (No. 2019024), and the Hebei University Science and Technology Research Project (No. ZD2019139).

## Conflict of interest

The authors declare that the research was conducted in the absence of any commercial or financial relationships that could be construed as a potential conflict of interest.

## Publisher's note

All claims expressed in this article are solely those of the authors and do not necessarily represent those of their affiliated organizations, or those of the publisher, the editors and the reviewers. Any product that may be evaluated in this article, or claim that may be made by its manufacturer, is not guaranteed or endorsed by the publisher.
